# Attenuating Cholinergic Transmission Increases the Number of Satellite Cells and Preserves Muscle Mass in Old Age

**DOI:** 10.3389/fnagi.2019.00262

**Published:** 2019-09-24

**Authors:** Sydney K. Vaughan, Natalia M. Sutherland, Gregorio Valdez

**Affiliations:** ^1^Department of Molecular Biology, Cell Biology and Biochemistry, Brown University, Providence, RI, United States; ^2^Fralin Biomedical Research Institute, Virginia Tech Carilion, Roanoke, VA, United States; ^3^Graduate Program in Translational Biology, Medicine, and Health, Virginia Tech, Blacksburg, VA, United States; ^4^Department of Biological Sciences, Virginia Tech, Blacksburg, VA, United States

**Keywords:** cholinergic transmission, VAChT, Pax7, aging, synapse, sarcopenia

## Abstract

In addition to driving contraction of skeletal muscles, acetylcholine (ACh) acts as an anti-synaptogenic agent at neuromuscular junctions (NMJs). Previous studies suggest that aging is accompanied by increases in cholinergic activity at the NMJ, which may play a role in neuromuscular degeneration. In this study, we hypothesized that moderately and chronically reducing ACh could attenuate the deleterious effects of aging on NMJs and skeletal muscles. To test this hypothesis, we analyzed NMJs and muscle fibers from heterozygous transgenic mice with reduced expression of the vesicular ACh transporter (VAChT; VKD^Het^), which present with approximately 30% less synaptic ACh compared to control mice. Because ACh is constitutively decreased in VKD^Het^, we first analyzed developing NMJs and muscle fibers. We found no obvious morphological or molecular differences between NMJs and muscle fibers of VKD^Het^ and control mice during development. In contrast, we found that moderately reducing ACh has various effects on adult NMJs and muscle fibers. VKD^Het^ mice have significantly larger NMJs and muscle fibers compared to age-matched control mice. They also present with reduced expression of the pro-atrophy gene, Foxo1, and have more satellite cells in skeletal muscles. These molecular and cellular features may partially explain the increased size of NMJs and muscle fibers. Thus, moderately reducing ACh may be a therapeutic strategy to prevent the loss of skeletal muscle mass that occurs with advancing age.

## Introduction

Neurotransmission is vital for proper communication across synapses in the central and peripheral nervous systems. Therefore, abnormal neurotransmission is associated with a variety of cognitive and motor deficits and numerous studies have been carried out with the goal of uncovering modalities to preserve and restore neurotransmission (Sarter et al., 2007; Campanari et al., 2016). At the neuromuscular junction (NMJ), the synapse between motor neurons and skeletal muscles, cholinergic transmission drives muscle contraction following the release of acetylcholine (ACh) and subsequent activation of nicotinic ACh receptors (nAChRs; Hughes et al., 2006). Additionally, ACh has been shown to play a critical role in sculpting the morphology of the postsynapse, the region of muscle fibers containing nAChR clusters and other NMJ-associated molecules. ACh affects the structural organization of adult NMJs by directly initiating the disassembly of nAChR clusters (Misgeld et al., 2002; Lin et al., 2005; An et al., 2010). For example, carbachol (CCh), a slowly hydrolyzing mimetic of ACh, causes the disassembly of spontaneously forming nAChR clusters on cultured myotubes (Misgeld et al., 2005). This action of CCh, however, is prevented when myotubes are also treated with neuronal-derived agrin (z-agrin), indicating that ACh and z-agrin have counteracting effects on the stability of NMJs *in vivo* (Misgeld et al., 2005).

Additional support for ACh acting as an anti-synaptogenic agent arose from the analysis of mice lacking the enzyme responsible for ACh synthesis, choline acetyltransferase (ChAT), and/or agrin. In ChAT null mice, the formation and subsequent stability of nAChR clusters at the postsynaptic region of the NMJ are similar to wild type mice (Misgeld et al., 2005). This is in stark contrast to mice lacking agrin, which acts to stabilize nAChR clusters. In agrin knockout mice, nAChR clusters disassemble upon the arrival of motor axons during the initial formation of the NMJ (Lin et al., 2005), suggesting that ACh released from motor axons causes the dispersal of nAChRs. However, the number of nAChR clusters is similar in mice lacking both ChAT and z-agrin compared to wild type mice during early developmental stages (Misgeld et al., 2005). Together, these findings demonstrate that ACh acts as an anti-synaptogenic factor while z-agrin functions to counteract the negative effects of ACh on NMJs. Thus, the level of ACh relative to z-agrin must be tightly regulated during development and in adulthood for the NMJs to properly develop and remain stable.

In recent years, data have been published indicating that ACh levels become dysregulated during normal aging and during the progression of amyotrophic lateral sclerosis (ALS). Specifically, in the gastrocnemius and diaphragm muscles, the amplitude of miniature endplate potentials (MEPPs), which represents the spontaneous release of vesicles at active zones, was found to be larger in old mice compared to young mice (Banker et al., 1983; Pousinha et al., 2015). The frequency of MEPPs was also found significantly increased in ALS-affected NMJs (Rocha et al., 2013; Arbour et al., 2015). Additionally, the frequency of spontaneous giant MEPPs (GMEPPs), which occurs when ACh is released from vesicles residing outside presynaptic active zones of NMJs, was shown to be increased in the diaphragm of old rodents (Pousinha et al., 2015). However, it is important to consider conclusions from a recent study that found no significant increase in mEPP or MEPC amplitude in muscles of old mice (Willadt et al., 2016), and thus more studies are necessary to ascertain the effect of aging on the cholinergic system specifically at NMJs. If aging NMJs have elevated ACh at the synaptic cleft, the area separating the presynapse and the postsynapse, its anti-synaptogenic actions would likely contribute to the degeneration of NMJs and atrophy of skeletal muscles that invariably occur with advancing age and during the progression of ALS (Valdez et al., 2010, 2012).

Our lab recently tested the hypothesis that a modest and persistent increase in ACh at the synaptic cleft destabilizes the NMJ. For this, we used transgenic mice with altered expression of the vesicular ACh transporter (VAChT; Prado et al., 2006; Kolisnyk et al., 2013). VAChT functions to load ACh into synaptic vesicles and thereby affects the amount of neurotransmitter released at the synaptic cleft (Prado et al., 2013). Mice overexpressing VAChT (*ChAT–ChR2–EYFP*, Kolisnyk et al., 2013) release more ACh at cholinergic synapses, resulting in a moderate and constitutive increase in MEPP amplitude (Sugita et al., 2016). Using these mice, we demonstrated that NMJs prematurely acquire age-related morphological features (Sugita et al., 2016). We also discovered that this genetic augmentation of ACh levels accelerates NMJ degeneration in the SOD1^G93A^ mouse model for ALS (Sugita et al., 2016). These effects were specific to adult mice since we found no evidence that increasing ACh levels affects developing NMJs and muscle fibers. The aforementioned findings indicate that reducing ACh levels may be a promising strategy for preventing degeneration of NMJs during normal aging and throughout the progression of ALS. However, ACh cannot be drastically reduced given its central function in initiating muscle contraction. To exemplify this point, studies have shown that reducing ACh levels at the synaptic cleft by 70% causes pathophysiological changes that resemble myasthenia gravis in mice (Prado et al., 2006; Rodrigues et al., 2013; Magalhães-Gomes et al., 2018).

In this study, we sought to determine the effect of moderately reducing levels of synaptic ACh by approximately 30% in developing, adult, and aging NMJs and muscle fibers. To test this, we used heterozygous VAChT-KD transgenic mice (VKD^Het^), which have decreased expression of VAChT that results in approximately 30% less ACh loaded and released from synaptic vesicles, and a concomitant decrease in the MEPP amplitude (Prado et al., 2006). The lower levels of ACh had no discernable effects on the development of NMJs and muscle fibers. However, reducing ACh increased the size of NMJs and muscle fibers in adult and aged mice compared to controls. Accompanying these morphological changes, skeletal muscles in adult VKD^Het^ mice express lower levels of the pro-atrophy gene, Foxo1, and have more satellite cells. Together, the data in this manuscript suggest that moderately decreasing ACh may slow the loss of skeletal muscle mass, known as sarcopenia, with advancing age.

## Materials and Methods

### Animals

Heterozygous VAChT-KD mice (Prado et al., 2006) were a generous gift of Dr. Marco Prado. Thy1-YFP16 (Feng et al., 2000) were a gift of Dr. Josh Sanes. Because homozygous VAChT-KD animals do not breed well, heterozygous VAChT-KD animals were bred with Thy1-YFP16 mice to generate heterozygous VAChT-KD mice expressing Thy1-YFP16 (herein referred to as VKD^Het^) and littermate Thy1-YFP16 controls. Genotyping was carried out using RT-PCR following proteinase K DNA extraction with subsequent purification using a QIAquick PCR purification kit (Qiagen 28104). All transgenic animals were allowed free access to food and water and housed with a 12-h light/dark cycle. Mice were anesthetized using isoflurane and either dissected immediately for fresh tissue or perfused transcardially with 1× PBS (pH 7.4) and 4% paraformaldehyde (PFA) for fixed tissue. All experiments were carried out under NIH guidelines and animal protocols approved by the Virginia Tech Institutional Animal Care and Use Committee.

### Imaging NMJs

The extensor digitorum longus (EDL) muscle was dissected from VKD^Het^ and littermate Thy1-YFP16 mice, washed three times in 1× PBS, and incubated for 2 h with Alexa 555 conjugated α-bungarotoxin (fBTX, Life Technologies; 1:1,000 in 1× PBS). After washing once more with 1× PBS, muscles were whole-mounted onto slides using Vectashield (Vector Labs).

### Intensity Analysis

NMJs from P9 EDL muscles were blocked for 1 h with 3% BSA, 5% goat serum, and 0.5% Triton-X. Samples were then incubated with primary anti-VAChT (Millipore; AB1588; 1:250) for 24 h at 4°C. After three washes with 1× PBS, samples were incubated with secondary antibody Alexa 488 goat anti-guinea pig (Life Technologies A11073; 1:1,000) and Alexa 555 conjugated α-bungarotoxin (fBTX, Life Technologies; 1:1,000) for 2 h at room temperature. Then samples were washed three additional times with 1× PBS and mounted using Vectashield (Vector Labs). Intensity analysis was done using ZEN software (Zeiss) with background correction.

### NMJ Analysis

To analyze structural features at NMJs, maximum intensity projections of confocal stacks were created using ZEN software (Zeiss). We analyzed structural features as previously described (Valdez et al., 2010). Briefly, fragmented nAChRs are defined as five or more separated islands of nAChR clusters. Full or partial denervation describes postsynaptic sites lacking the opposing nerve terminal. In developing NMJs, colocalization is a measure of overlap between the 488 and 555 fluorescence channels analyzed using ZEN software. Multiple innervation is the simultaneous innervation of a postsynapse by two or more axons. To quantify the area of NMJs, the area of the region occupied by nAChRs, labeled by fBTX, was measured using ImageJ software. At least 25 NMJs were analyzed from each muscle to represent an individual mouse. At least three animals per genotype were analyzed to generate the represented data.

### TA Sectioning

TA muscle was dissected from perfused mice and incubated in 30% sucrose for 48 h, then cut in half at the largest diameter and placed in a 10 × 10 × 5 mm Cryomold with Tissue Freezing Medium (Triangle Biomedical Science, Inc., Durham, NC, USA) and cut in 16 μm slices using a cryostat.

### Muscle Fiber Diameter/Central Nuclei

The sections were stained with Alexa-488-conjugated wheat germ agglutinin (WGA; 1:700) and 4′,6-diamidino-2-phenylindole (DAPI: Sigma-Aldrich; 28718-90-3; 1:1,000) diluted in 1× PBS for 2 h at room temperature. The sections were then washed three times in 1× PBS and mounted using Vectashield. Images were acquired using a Zeiss LSM 700 confocal microscope and maximum intensity projections from z-stacks were created with Zen Black (Zeiss). The area outlined by WGA was measured and Feret’s diameter was determined using ImageJ. At least 100 muscle fibers per cross-section were randomly selected for analysis. From the selected fibers, the percent of muscle fibers with centrally located myonuclei was recorded. At least three cross-sectional images were analyzed and averaged together to represent an individual mouse. At least three animals per genotype were analyzed to generate the represented data.

### Muscle Cross-Sectional Area

The sections were stained with Alexa-488-conjugated (WGA; 1:700) and DAPI (Sigma-Aldrich; 28718-90-3; 1:1,000) diluted in 1× PBS for 2 h at room temperature. The sections were then washed three times in 1× PBS and mounted using Vectashield. Images were acquired using a Zeiss LSM 700 confocal microscope and maximum intensity projections from z-stacks were created with Zen Black (Zeiss). The total cross-sectional area (CSA) of the TA muscles was outlined by WGA and measured using ImageJ. The average CSA was calculated from at least three cross-sectional images per individual animal. At least three animals per experimental group were analyzed to generate the represented data.

### Pax7 immunostaining

An antibody against Pax7, obtained as a gift from Dr. Julia Von Maltzahn, was used according to the protocol described in Ahrens et al. (2018). In brief, sections were washed, permeabilized using 0.1% Triton-X and 0.1 M glycine in 1× PBS, and then blocked with Mouse on Mouse blocking reagent (Vector Labs, MKB-2213) at a 1:40 dilution in 1× PBS for 1 h at room temperature. Sections were then incubated in anti-Pax-7 and rabbit anti-laminin (Sigma L9393; 1:300) overnight at 4°C. After washing, sections were incubated in secondary antibodies diluted 1:1,000 in 5% horse serum in 1× PBS. Secondary antibodies used were Alexa-555 mouse IgG1 and Alexa-488 donkey anti-rabbit (Life Technologies). Muscles were then incubated in DAPI (1:1,000) and mounted using Vectashield. Maximum intensity projections were acquired using a Zeiss LSM 700 confocal microscope with Zen Black (Zeiss). Pax7-positive nuclei were identified as nuclei double-labeled with Pax7 antibody and DAPI. The percentage of Pax7-positive nuclei was calculated as the number of positive nuclei per total number of muscle fibers per muscle and is represented relative to 4-month control muscle. At least 1,000 muscle fibers were analyzed per animal. The average number of muscle fibers per TA muscle cross-section was not significantly different between genotypes and ages (4 month control = 1,267 ± 460, 4 month VKD^Het^ = 1,775 ± 156, 17 month control = 1,596 ± 147, 17 month VKD^Het^ = 1621 ± 356).

### Expression Analysis Using Quantitative PCR (qPCR)

TA and EDL muscles and spinal cord were dissected and flash-frozen in liquid nitrogen. Total RNA was prepared using Aurum Total RNA Mini kit (Bio-Rad) following the manufacturer’s instructions. cDNA was synthesized from 500 ng of total RNA using the iScript cDNA synthesis kit (Bio-Rad) and subsequently diluted to 100 ng. PCR amplification was performed on the Bio-Rad CFX Connect Real-Time System (Bio-Rad) using iTaq Universal SYBR Green Supermix (Bio-Rad). All primers used in this study are listed in Supplementary Table S1.

### Statistical Analysis

Student’s *t*-tests were used to compare differences between two groups. For statistical analysis of multiple groups, a one-way ANOVA with *post hoc* Bonferroni test was used to determine significance. A Kolmogorov–Simirnov test was used to compare differences between sample distributions. Data are expressed as the mean ± SEM (standard error measurement) with individual data points represented along the error bar. *P* < 0.05 was considered statistically significant. Statistical analysis was performed using GraphPad Prism software.

## Results

### VAChT Is Decreased in the Spinal Cord and at NMJs of VKD^Het^ Mice

**Figure 1:**
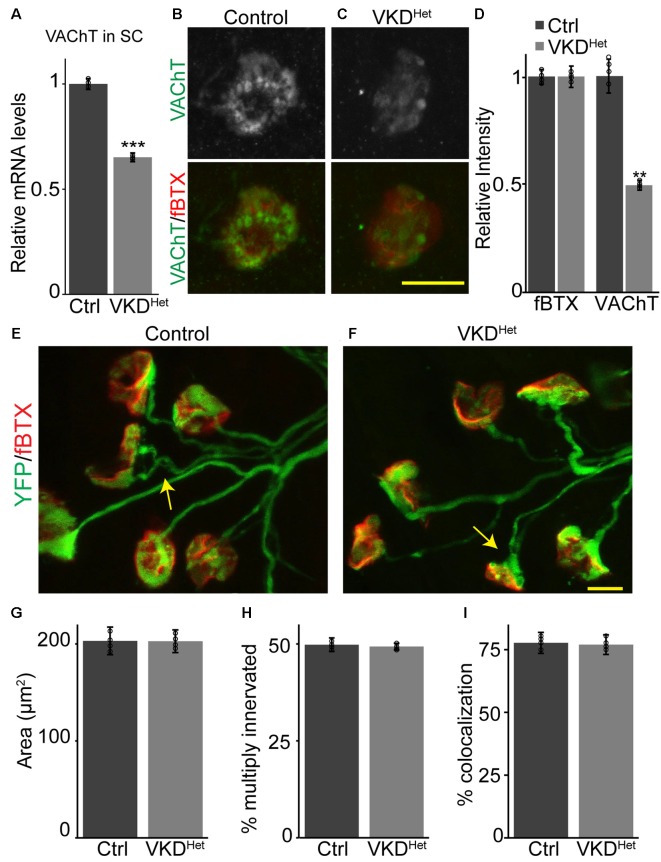
Reduced expression of vesicular acetylcholine transporter (VAChT) has no impact on neuromuscular junction (NMJ) development. Expression of VAChT mRNA is significantly reduced in the spinal cord of adult VKD^Het^ mice **(A)**. VAChT protein was visualized at NMJs in extensor digitorum longus (EDLs) from p9 control **(B)** and VKD^Het^ **(C)** mice using anti-VAChT (green) and α-bungarotoxin (red). The intensity of VAChT protein at the NMJ is significantly reduced in VKD^Het^ mice compared to controls **(D)**. NMJs were visualized in EDL muscles from p9 control **(E)** and VKD^Het^ **(F)** mice using yellow fluorescence protein (YFP; green) and α-bungarotoxin (red). The average area of receptor plaques is unchanged in VKD^Het^ mice compared to controls **(G)**. The percentage of multiply innervated NMJs (arrows) is also similar between control and VKD^Het^ mice **(H)**. Colocalization between pre- and post-synapse is at similar levels in control and VKD^Het^ mice **(I)**. Represented as mean ± SEM. Expression is normalized to GAPDH and relative to control. Scale bars = 10 μm. Only male littermates were used for these experiments. **(A)** Control *n* = 3; VKD^Het^ *n* = 3; 13 months old, **(B–I)** control *n* = 4; VKD^Het^ *n* = 4; 9 days old. At least 25 NMJs were examined per animal. Black circles represent individual data points. **p < 0.01, ***p < 0.001.

To determine the impact of moderately and chronically reducing synaptic ACh on NMJs and muscle fibers, we utilized a transgenic mouse line with reduced expression of the VAChT gene, known as VAChT-KD mice (Prado et al., 2006). In this transgenic mouse line, VAChT expression was shown to be reduced by approximately 65% in homozygous and 45% in heterozygous mice (Prado et al., 2006). In this study, we only examined heterozygous VAChT-KD mice, herein referred to as VKD^Het^, to determine the impact of moderately and chronically reducing synaptic ACh on NMJs and muscle fibers. First, we validated previous findings showing that VAChT mRNA is significantly lower (−34.83 ± 2.092%) in the spinal cord of VKD^Het^ mice compared to litter mate control mice (Figure 1A). We then asked if VAChT protein is reduced specifically at NMJs of VKD^Het^ mice. To visualize VAChT protein at NMJs, we used an antibody against VAChT together with fluorescently tagged α-bungarotoxin (fBTX), which binds selectively to nAChRs located on the postsynaptic region of the NMJ. Quantification of fluorescence intensity revealed a marked reduction in VAChT (−53.5 ± 7.63%) but not fBTX at NMJs of the EDL muscle of VKD^Het^ mice compared to control mice (Figures 1B–D). These data demonstrate that VAChT transcripts and protein are reduced in the spinal cord and at NMJs, respectively, in VKD^Het^ mice.

### Normal Development of NMJs and Muscle Fibers in VKD^Het^ Mice

Cholinergic transmission has been postulated to play a central role in the timely development of NMJs and muscle fibers in animals (Misgeld et al., 2002, 2005; Lin et al., 2005). We, therefore, hypothesized that moderately decreasing ACh levels, and thereby reducing cholinergic transmission, would alter the rate of NMJ and muscle fiber development. We generated VKD^Het^ and control mice expressing yellow fluorescence protein (YFP) specifically in neurons, including alpha-motor neurons (Feng et al., 2000), to better visualize motor nerve endings and thus assess the innervation status of NMJs. We examined NMJs from the EDL muscle at postnatal day 9 (P9), an age when synaptic elimination is nearly complete and NMJs are transitioning from a small plaque to a large pretzel-like structure in mice (Sanes and Lichtman, 1999; Shi et al., 2012). We found no obvious difference in the overall morphology of NMJs between P9 VKD^Het^ and littermate control mice (Figures 1E,F). The size of nAChR clusters, the incidence of multiply innervated postsynaptic sites, and the percent apposition between pre- and post-synaptic sites are all unchanged in VKD^Het^ compared to control mice (Figures 1G–I).

During development, the NMJ also undergoes molecular and functional changes that increase the fidelity of cholinergic transmission. In particular, it is well-established that the gamma subunit of nAChRs is replaced by the epsilon subunit as NMJs mature (Yumoto et al., 2005; Millar and Harkness, 2008), a shift that alters the functional properties of nAChR pentamers (Mishina et al., 1986; Brehm, 1989; Naguib et al., 2002). To determine the impact of reducing ACh on the functional maturation of NMJs, we examined the expression of the gamma and epsilon subunits in P9 TA and EDL muscles. This analysis showed that both the gamma and epsilon subunits are expressed at similar levels in VKD^Het^ compared to control mice at P9 (Supplementary Figure S1), supporting the morphological findings above showing that developing NMJs in VKD^Het^ mice are indistinguishable from those in control mice.

We then examined the impact of reducing ACh levels on the maturation and differentiation of skeletal muscle fibers into unique functional types. There are four major types of skeletal muscle fibers that can be identified based on their expression of myosin heavy chain (MyHC) isoforms (type 1, 2A, 2X or 2B; Agbulut et al., 2003; Kaasik et al., 2012). We found no difference in mRNA levels for MyHC type 1, 2A, 2B and 2X in the TA and EDL muscles between P9 VKD^Het^ and control mice (Supplementary Figure S1). Collectively, these findings strongly suggest that moderately reducing synaptic ACh does not affect the development of NMJs and muscle fibers in the TA and EDL muscles.

### NMJs Are Larger in Adult and Aged VKD^Het^ Mice

**Figure 2:**
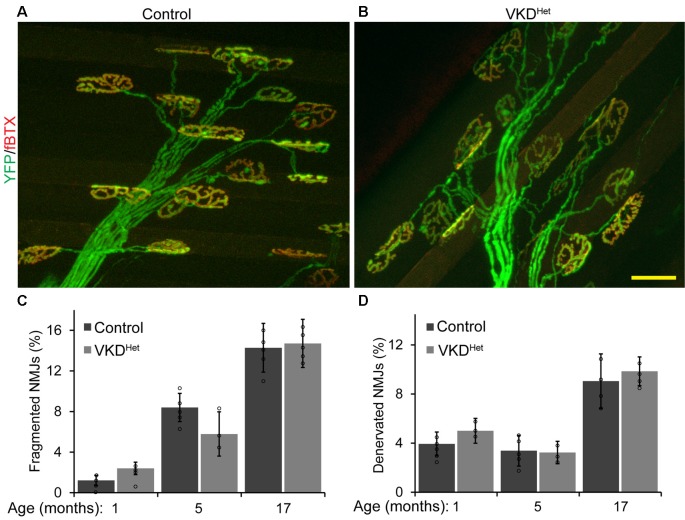
Incidence of fragmented and denervated NMJs are unchanged in adult and aged VKD^Het^ mice. NMJs were visualized in EDL muscles from 1, 5, and 17 month-old control **(A)** and VKD^Het^ **(B)** mice using YFP (green) and α-bungarotoxin (red). The percentage of fragmented NMJs is similar between control and VKD^Het^ mice at each age examined **(C)**. The percentage of fully denervated NMJs is similar between control and VKD^Het^ mice at each age examined **(D)**. Represented as mean ± SEM. Scale bar = 20 μm. Representative images are from 5 month-old mice. Only male mice were used for these experiments. Control *n* = 3; VKD^Het^ *n* = 3 for each age group. At least 50 NMJs were analyzed per animal. Black circles represent individual data points.

**Figure 3:**
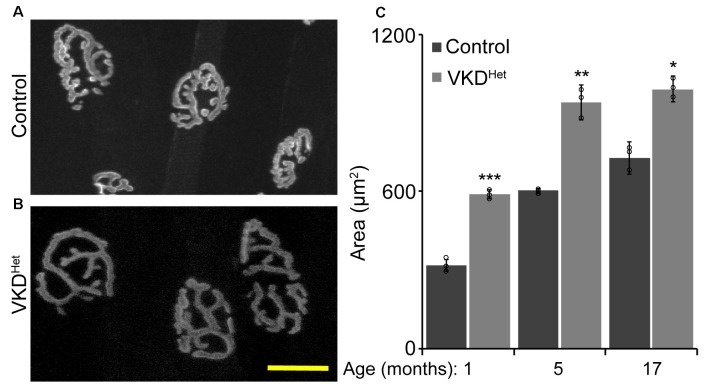
Increased NMJ area in VKD^Het^ muscle. Nicotinic Ach receptors (nAChRs) were visualized in EDL muscles from 1, 5, and 17 month-old control **(A)** and VKD^Het^ **(B)** mice using α-bungarotoxin. In each age group, nAChRs from VKD^Het^ mice have a significantly larger area compared to controls **(C)**. Represented as mean ± SEM. Scale bar = 20 μm. Representative images are from 5 month-old mice. Only male mice were used for these experiments. Control *n* = 3; VKD^Het^ *n* = 3 for each age group. At least 50 NMJs were analyzed per animal. Black circles represent individual data points. *p < 0.05, **p < 0.01, ***p < 0.001.

Published findings indicate that dysregulated and increased release of ACh may contribute to degeneration of NMJs with advancing age and during the progression of ALS (Sugita et al., 2016). We hypothesized that moderately reducing ACh levels would have the opposite effect, and instead slow aging of NMJs. To test this possibility, we assessed the impact of decreasing synaptic ACh on NMJs in the EDL muscle of 1, 5, and 17-month-old VKD^Het^ and aged-matched control mice. We examined NMJs for fragmentation and denervation, two cellular features prevalent in aged NMJs in the EDL muscle of mice (Valdez et al., 2010). We found no significant differences in the incidence of fragmented and denervated NMJs between VKD^Het^ and control mice at all ages examined (Figures 2A–D). In addition to fragmentation and denervation, NMJs change in size with advancing age (Cheng et al., 2013). Smaller NMJs are present in atrophying, newly regenerated, or healthy slow-twitch (Type I) muscle fibers (Seene et al., 2017). Larger NMJs, on the other hand, are present on healthy fast-twitch (Type II) muscle fibers (Seene et al., 2017). We found that, on average, NMJs are significantly larger in 1-, 5-, and 17-month-old VKD^Het^ compared to age-matched control mice (Figures 3A–C). This finding is not surprising since nAChR clusters are expected to be more stable when ACh is reduced (Misgeld et al., 2002; Lin et al., 2005; An et al., 2010).

To complement morphological analysis of NMJs in adult mice, we examined the impact of reducing ACh on the expression of genes with critical roles in the structural and functional characteristics of NMJs. We found no difference in mRNA levels for the gamma and epsilon nAChR subunits, muscle specific kinase (MuSK), cyclin dependent kinase 5 (CDK5), docking protein 7 (Dok7), LDL receptor related protein 4 (LRP4), and Rapsyn in the TA and EDL muscles of 4- and 17-month-old VKD^Het^ mice compared to control mice (Supplementary Figure S2). Unexpectedly, this analysis revealed that acetylcholinesterase (AChE) is significantly higher in 17-month-old VKD^Het^ mice compared to age-matched control mice (Supplementary Figure S2). We next explored the possibility that reduction of VAChT is accompanied by changes in expression of other genes with critical roles in cholinergic transmission and in stabilizing the NMJ in motor neurons. We examined the ChAT, critical for the synthesis of ACh, and z-agrin isoforms in the spinal cord of VKD^Het^ mice and control mice. We found transcripts for ChAT and all three z-agrin isoforms levels unchanged in the spinal cord of adult VKD^Het^ compared to control mice (Supplementary Figure S3). This is not surprising given that other studies have not detected changes in *z*-agrin isoform levels between aged and young control mice (Samuel et al., 2012).

### Muscle Fibers Are Larger in Adult and Aged VKD^Het^ Mice

**Figure 4:**
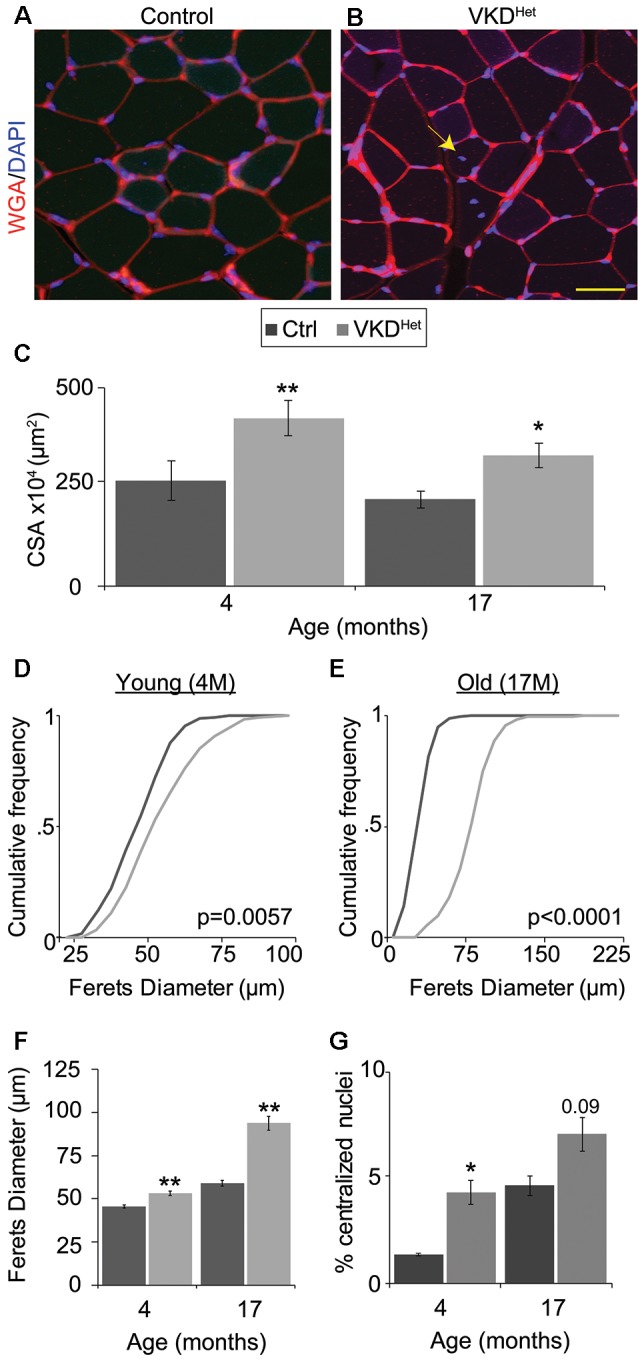
Increased fiber area in VKD^Het^ mice. Muscle fibers of TA muscle from control **(A)** and VKD^Het^ **(B)** mice were visualized using wheat germ agglutinin (WGA; red) and DAPI (blue). The average cross-sectional area (CSA) is significantly increased in VKD^Het^ muscles at 4 and 17 months of age **(C)**. The cumulative distribution of muscle fiber area in VKD^Het^ mice is significantly different from controls at 4 **(D)** and 17-months of age **(E)**. The average fiber area is also increased **(F)** and there are more centralized myonuclei (arrow) in VKD^Het^ mice **(G)**. Represented as mean ± SEM. Scale bar = 20 μm. Only male mice were used for these experiments. Representative images are from 4 month-old mice. Control *n* = 4; VKD^Het^ *n* = 4 in each age group. At least 100 fibers were analyzed per animal. Black circles represent individual data points. *p < 0.05, **p < 0.01.

The increased size of NMJs in adult VKD^Het^ mice suggested that reducing ACh may have a similar effect on muscle fibers. To visualize and measure the area of muscle fibers, we stained 16 μm muscle cross-sections from the TA muscle of 4- and 17-month-old mice with WGA (Figures 4A,B). We found that the average muscle fiber CSA is larger in 4- and 17-month-old VKD^Het^ mice compared to age-matched control mice (Figure 4C). This increase in the average muscle fiber CSA in VKD^Het^ mice may be due to an increase in the diameter of individual fibers, an increase in the number of muscle fibers, or a combination of the two. To distinguish between these possibilities, we used the Feret’s Diameter analysis to determine the average size of individual muscle fibers. A cumulative frequency histogram and a two-sample Komogorov-Smirnov test revealed an increase in large muscle fibers in the TA muscle of 4- and at 17-month-old VKD^Het^ mice compared to control mice of the same age and sex (Figures 4D,E). Not surprisingly, the average Feret’s Diameter of individual muscle fibers is significantly larger in the TA muscle of 4- and 17-month-old VKD^Het^ mice compared to control mice of the same age (Figures 4F,G). However, we found no significant differences in mRNA levels for MyHC type 1, 2A, 2B and 2X in the TA and EDL muscles between 4 or 17-month-old VKD^Het^ and control mice (Supplementary Figure S4).

The increased size of muscle fibers in VKD^Het^ mice may result from ongoing fusion, and thus the addition, of myoblasts with matured muscle fibers. If this is true, muscle fibers in VKD^Het^ mice should contain more centralized myonuclei, a hallmark of either regenerating or growing muscle fibers (Folker and Baylies, 2013). To examine this possibility, we examineed the location of myonuclei in muscle cross-sections labeled with DAPI. In 4-month-old VKD^Het^, the incidence of muscle fibers with centralized myonuclei is significantly higher compared to control mice (Figure 4F). The fact that VKD^Het^ animals have larger muscle fibers, and have increased centralized myonuclei, indicates that reducing ACh levels promotes the fusion of myoblasts with existing muscle fibers. These findings suggest that decreasing levels of ACh promotes myogenesis, resulting in larger muscle fibers in adult mice.

### Satellite Cells Are More Abundant in Old VKD^Het^ Mice

**Figure 5:**
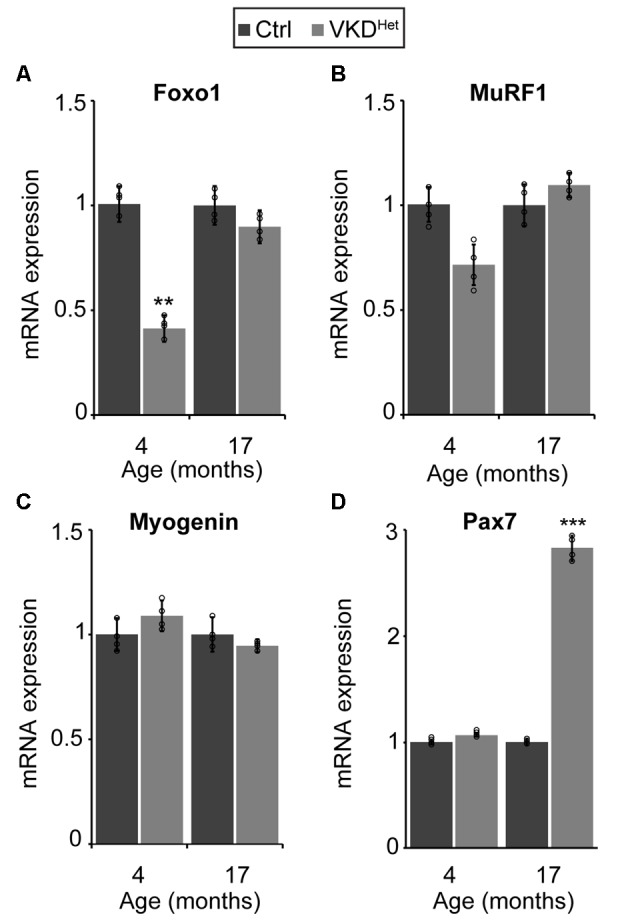
Molecular changes in adult VKD^Het^ muscle. At 4 months of age, there is a significant down regulation of FOXO-1 in VKD^Het^ mice **(A)**. While Muscle RING-Finger Protein 1 (MuRF1; **B**) and myogenin **(C)** expression is unchanged, Pax7 expression is significantly upregulated in 17 month-old VKD^Het^ mice **(D)**. Represented as mean ± SEM. Expression is normalized to GAPDH and relative to control. RNA isolated from TA muscles. Representative images from 17 month-old samples. Scale bar = 50 μm. Only male mice were used for these experiments. Control *n* = 4; VKD^Het^ *n* = 4 in each age group. Black circles represent individual data points. **p < 0.01, ***p < 0.001.

**Figure 6:**
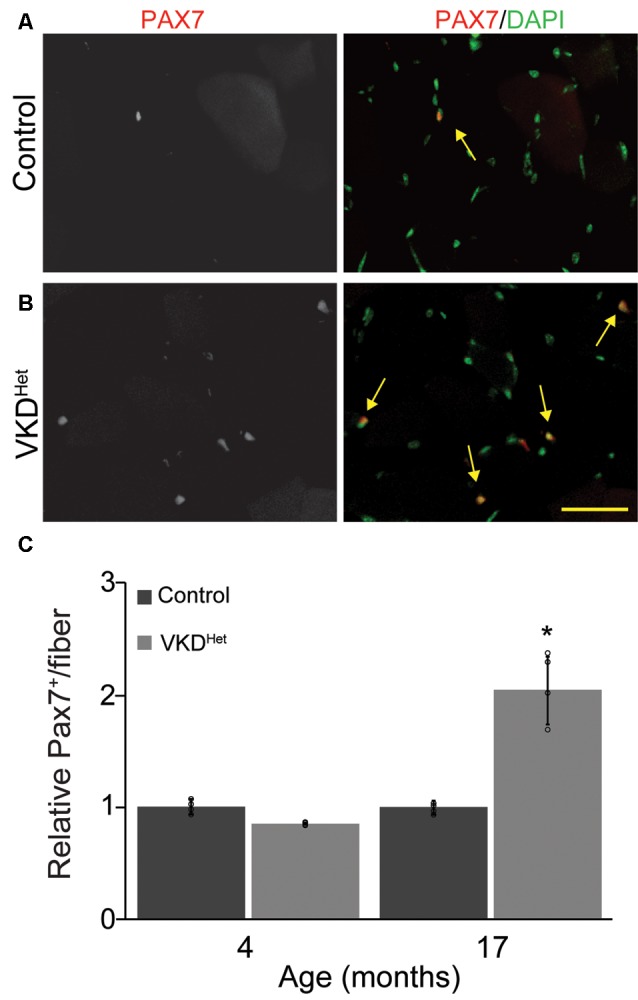
Increased Pax7 nuclei in VKD^Het^ muscle. TA cross-sections from control **(A)** and VKD^Het^ **(B)** mice were labeled with Pax7 (red) and DAPI (green). While the percentage of muscle fibers with Pax7+ nuclei is unchanged at 4 months of age, there is a significant increase in the percentage of Pax7+ nuclei in 17 month-old VKD^Het^ mice compared to controls **(C)**. Represented as mean ± SEM. Representative images from 17 month-old samples. Scale bar = 50 μm. Only male mice were used for these experiments. Control *n* = 4; VKD^Het^ *n* = 4 in each age group. Black circles represent individual data points. *p < 0.05.

The expression of pro-atrophy genes and the presence of satellite cells have been shown to correlate with muscle fiber size (Kamei et al., 2004; Murach et al., 2018). We examined the expression of two pro-apoptotic genes, Forkhead box protein O1 (Foxo1) and Muscle RING-Finger Protein 1 (MuRF-1; Gomes et al., 2001; Sandri et al., 2004; Apel et al., 2009; Natanek et al., 2013), in skeletal muscles of adult VKD^Het^ mice. We found Foxo1 mRNA significantly reduced in the TA and EDL muscles of 4-month-old VKD^Het^ mice (Figure 5A). However, MuRF1 expression was unchanged in VKD^Het^ mice compared to control mice (Figure 5B). These findings suggest that reduced expression of Foxo1, a prominent pro-atrophy gene, partly accounts for the increased size of muscle fibers in young adult VKD^Het^ mice. However, other molecular mechanisms likely contribute to the increased muscle fiber size found in VKD^Het^ mice, since Foxo1 is expressed at similar levels in 17-month-old VKD^Het^ compared to control mice of the same age. We, therefore, examined the expression of myogenin and Pax7, two genes important for myogenesis, in VKD^Het^ mice. We found myogenin expression unchanged in VKD^Het^ compared to control mice at 4 and 17 months of age (Figure 5C). Pax7 expression is also unchanged in 4-month-old VKD^Het^ mice compared to age-matched control mice (Figure 5D). However, we found Pax7 significantly increased in the TA muscle of 17-month-old VKD^Het^ mice (Figure 5D). To determine if the increased level of Pax7 is a consequence of more satellite cells in aged VKD^Het^ mice, we stained cross-sections from the TA muscle with antibody against Pax7. In the TA muscle of 4-month-old VKD^Het^ mice, the number of Pax7-positive cells is unchanged compared to control mice of the same age. However, there are significantly more Pax7-positive cells in 17-month-old VKD^Het^ mice compared to control mice of the same age (Figures 6A–C). These findings show that reducing ACh increases the number of satellite cells in aged skeletal muscles.

## Discussion

Evidence continues to accrue suggesting that aberrant cholinergic activity contributes to neuromuscular degeneration associated with aging and ALS (Arbour et al., 2015;Pousinha et al., 2015). Importantly, our group showed that increasing the amount of ACh released at the synaptic cleft accelerates the appearance of aging and ALS-related pathological features at NMJs (Sugita et al., 2016). These findings, together with other studies showing that ACh on its own acts to disassemble the postsynaptic region of the NMJ (Misgeld et al., 2002; Lin et al., 2005; An et al., 2010), raised the prospect that moderately reducing ACh levels, without impeding muscle contraction, could have beneficial effects on NMJs and elsewhere in skeletal muscles. To test this hypothesis, we examined heterozygous transgenic mice (VKD^Het^) presenting with reduced expression of the VAChT, and thus decreased loading of ACh into synaptic vesicles. VKD^Het^ mice present with a mild reduction in MEPP amplitude (Prado et al., 2006), insufficient to affect overall neuromuscular function. This is due to the large safety factor of the NMJ, which allows it to retain the capacity to drive muscle contraction when ACh levels moderately decrease (Wood and Slater, 2001).

We found that NMJs and muscle fibers are indistinguishable in VKD^Het^ compared to control mice during development. There is no difference in the size of NMJs and of muscle fibers between genotypes. Additionally, the number of multiply innervated postsynaptic sites and the overlap with presynaptic sites is unchanged at NMJs of developing VKD^Het^ mice. Several factors may explain the normal development of NMJs and muscle fibers exposed to reduced ACh. For one, the amount of ACh in synaptic vesicles and released at the synaptic cleft is much higher during development (Takahashi et al., 2002). Thus, moderately reducing ACh may not discernably affect the size of nAChR clusters nor sufficiently diminish cholinergic activity to prolong synapse elimination (Ribchester and Taxt, 1983; Thompson, 1985; Ribchester, 1988).

In stark contrast, decreasing ACh markedly affected adult NMJs and muscle fibers. In young adult and aged VKD^Het^, NMJs are significantly larger compared to age-matched control mice. However, the incidence of fragmented and denervated NMJs was unchanged in aged VKD^Het^ mice. Additionally, several NMJ-associated genes remained expressed at similar levels in the spinal cord and in skeletal muscles of young adult and aged VKD^Het^ mice. The only exception is AChE, which we found significantly increased in skeletal muscles of aged VKD^Het^ mice. The two most parsimonious explanations for these disparate effects of reducing ACh on NMJ size and levels of NMJ-associated genes are: (1) the increased size of the NMJ may be a consequence of reduced internalization of nAChRs, thus leading to larger postsynaptic sites. In this case, muscle fibers may not be under pressure to alter the expression of nAChR subunits since the moderate loss of ACh may simply cause nAChR subunits to redistribute. For example, decreasing ACh may cause nAChR subunit protein to accumulate at the peripheral membrane resulting in decreased levels in vesicles destined for degradation or recycling. (2) The increased expression of AChE may result from skeletal muscles attempting to maintain AChE density to account for the expanded size of NMJs. In support of this hypothesis, AChE has been shown to increase in differentiating and thus growing muscle fibers (Fuentes and Taylor, 1993), which invariably leads to larger NMJs.

We also found that muscle fibers are larger in young adult and aged VKD^Het^ mice compared to age-matched control mice. In young adult VKD^Het^ mice, we found that reduced expression of Foxo1 contributes to the increased size of muscle. In aged VKD^Het^ mice, we found that increased expression of Pax7 and an associated increase in the number of satellite cells contributes to the increased size of muscle fibers. This finding is significant as it demonstrates that a moderate decrease in ACh slows sarcopenia, or the atrophy of skeletal muscles with advancing age, by increasing the number of satellite cells. However, our study does not address whether the effects of reducing ACh on satellite cells is direct or indirect. It is possible that ACh acts in both manners given that satellite cells have been shown to respond to changes in activity patterns (Tatsumi et al., 2006) and to increase in number under conditions that positively affect aging skeletal muscles (Krause et al., 1995; Tatsumi et al., 2001; Liu et al., 2017). Our study also does not address the possibility that reducing ACh levels alters the function of other molecular mechanisms with important roles in muscle fiber growth and maintenance.

This study demonstrates that moderately, chronically, and ubiquitously reducing ACh can mitigate the deleterious effects of aging on NMJs and muscle fibers. However, the use of a transgenic mouse line (Prado et al., 2006), with germ-line, global, and chronic reduction of VAChT expression, leaves open the possibility that temporally and spatially modulating the cholinergic system may further attenuate aging of NMJs and skeletal muscles. For example, moderately reducing ACh levels may be most beneficial immediately preceding or at the onset of aberrant changes in the cholinergic system caused by aging. Such temporal intervention for altering ACh levels at NMJs is possible using small molecules, such as vesamicol (Rodrigues et al., 2013). Additionally, it remains possible that other components of the cholinergic system could become dysregulated at the presynaptic and postsynaptic regions of the NMJ, independently of changes in ACh levels. Thus, it may be necessary to devise combinatorial strategies to modulate the cholinergic system in other resident cells in skeletal muscle to confer maximum protection to NMJs and muscle fibers from aging. This could include, but is not limited to, perisynaptic Schwann cells and macrophages (Pavlov et al., 2003; Kharraz et al., 2013; Lee and Vazquez, 2013; Ko and Robitaille, 2015).

Additionally, it remains possible that other components of the cholinergic system could become dysregulated at the presynaptic and postsynaptic regions of the NMJ, in addition to Terminal Schwann cells, independently of changes in ACh levels. In addition to cellular constituents of the NMJ, it may be necessary to devise combinatorial strategies to modulate the cholinergic system in other skeletal muscle resident cells to confer additional protection to NMJs and muscle fibers from aging. This could include, but is not limited to, activating the alpha7 nAChRs in macrophages to reduce the adverse effects of inflammation (Lee and Vazquez, 2013) while decreasing levels of ACh in synaptic vesicles. These and other potential combinatorial approaches aimed at maintaining the normal function of the cholinergic system could help preserve skeletal muscles and their NMJs into old age.

## Ethics Statement

All experiments were carried out under NIH guidelines and animal protocols approved by the Virginia Tech Institutional Animal Care and Use Committee.

## Author Contributions

SV and NS contributed to data collection, data analysis, and writing the manuscript. GV designed the study, prepared the manuscript, and procured funding. All authors read and approved the manuscript.

## Conflict of Interest

The authors declare that the research was conducted in the absence of any commercial or financial relationships that could be construed as a potential conflict of interest.
