# Supplementary material for: Attenuating Cholinergic Transmission Increases the Number of Satellite Cells and Preserves Muscle Mass in Old Age
Source: Front Aging Neurosci. 2019 Sep 24;11:262. doi: 10.3389/fnagi.2019.00262 (PMC6768977; doi:10.3389/fnagi.2019.00262)
Supplement: Supplementary file 1 [file Data_Sheet_1.PDF]

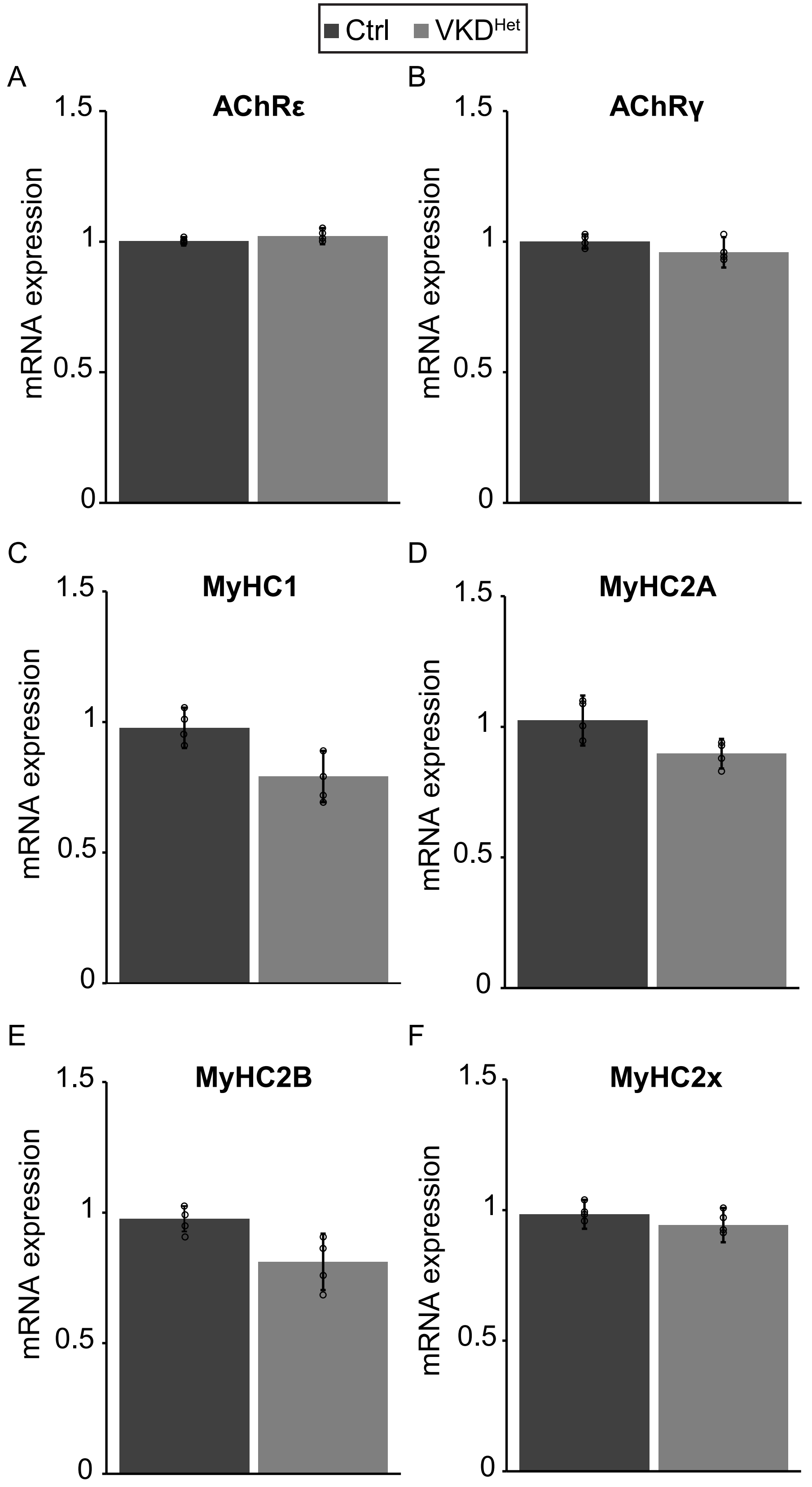

Ctrl
VKD^Het
A
AChRε
B
AChRγ
C
MyHC1
D
MyHC2A
E
MyHC2B
F
MyHC2x
mRNA expression
1.5
1
0.5
0

Supplementary Figure 1. Normal molecular profile at the NMJ of developing VKD^{Het} muscle. Expression of AChRε (A) and AChRγ (B) subunits are similar between control and VKD^{Het} mice. There are no significant changes in the expression of MyHC1 (C), MyHC2A (D), MyHC2B (E), or MyHC2x (F) between control and VKD^{Het} mice. Represented as mean ± SEM. Expression is normalized to GAPDH and relative to control. RNA isolated from TA and EDL muscles. Only male littermates were used for these experiments. Control n=4; VKD^{Het} n=4; 9 days old.

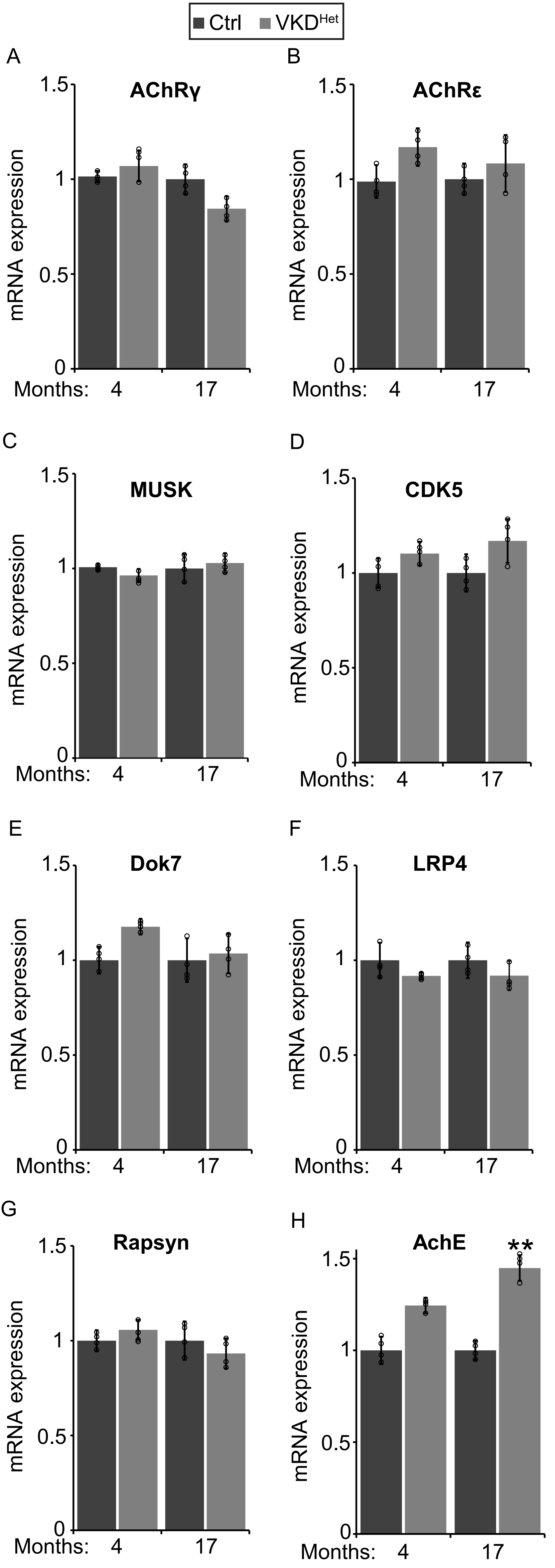
Ctrl
VKD^Het
A
AChRγ
B
AChRε
C
MUSK
D
CDK5
E
Dok7
F
LRP4
G
Rapsyn
H
AchE
**
mRNA expression
Months:
4
17
0
0.5
1
1.5

Supplementary Figure 2. AChE expression is increased in aging VKD^{Het} NMJs. There are no significant changes in the expression of other NMJ-associated proteins including AChRγ (A), AChRε (B), MUSK (C), CDK5 (D), Dok7 (E), LRP4 (F), or Rapsyn (G). However, expression of acetylcholinesterase is significantly upregulated in 17 month-old VKD^{Het} mice compared to controls (H). Represented as mean ± SEM. Expression is normalized to GAPDH and relative to control. RNA isolated from TA muscles. Only male mice were used for these experiments. Control n=4; VKD^{Het} n=4 in each age group. P-value= **<0.01.

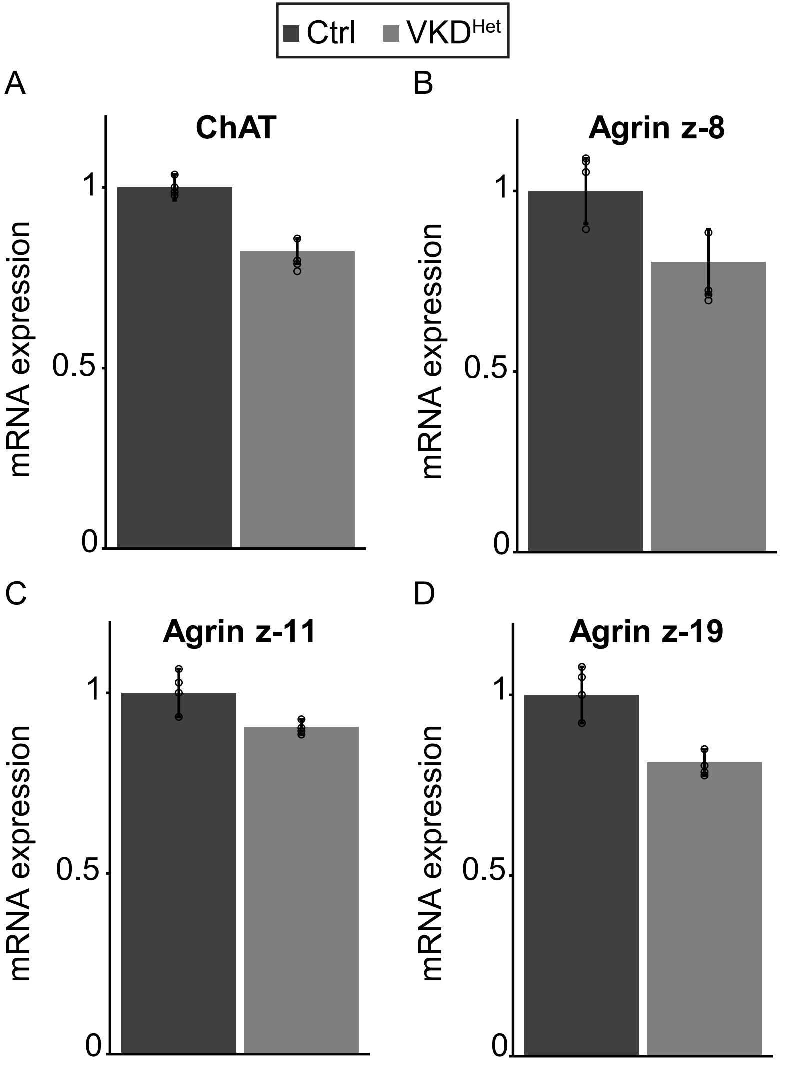

Ctrl
VKD^{Het}
A
ChAT
mRNA expression
1
0.5
0
B
Agrin z-8
mRNA expression
1
0.5
0
C
Agrin z-11
mRNA expression
1
0.5
0
D
Agrin z-19
mRNA expression
1
0.5
0

Supplementary Figure 3. Stable expression of nerve-derived factors in VKD^{Het} mice. The expression of ChAT remains unchanged in VKD^{Het} mice compared to controls (A). There are also no significant changes in the expression of three agrin isoforms in VKD^{Het} mice (B-D). Represented as mean ± SEM. Expression is normalized to GAPDH and relative to control. RNA isolated from spinal cord. Only 13 month-old male mice were used for these experiments. Control n=4; VKD^{Het} n=4.

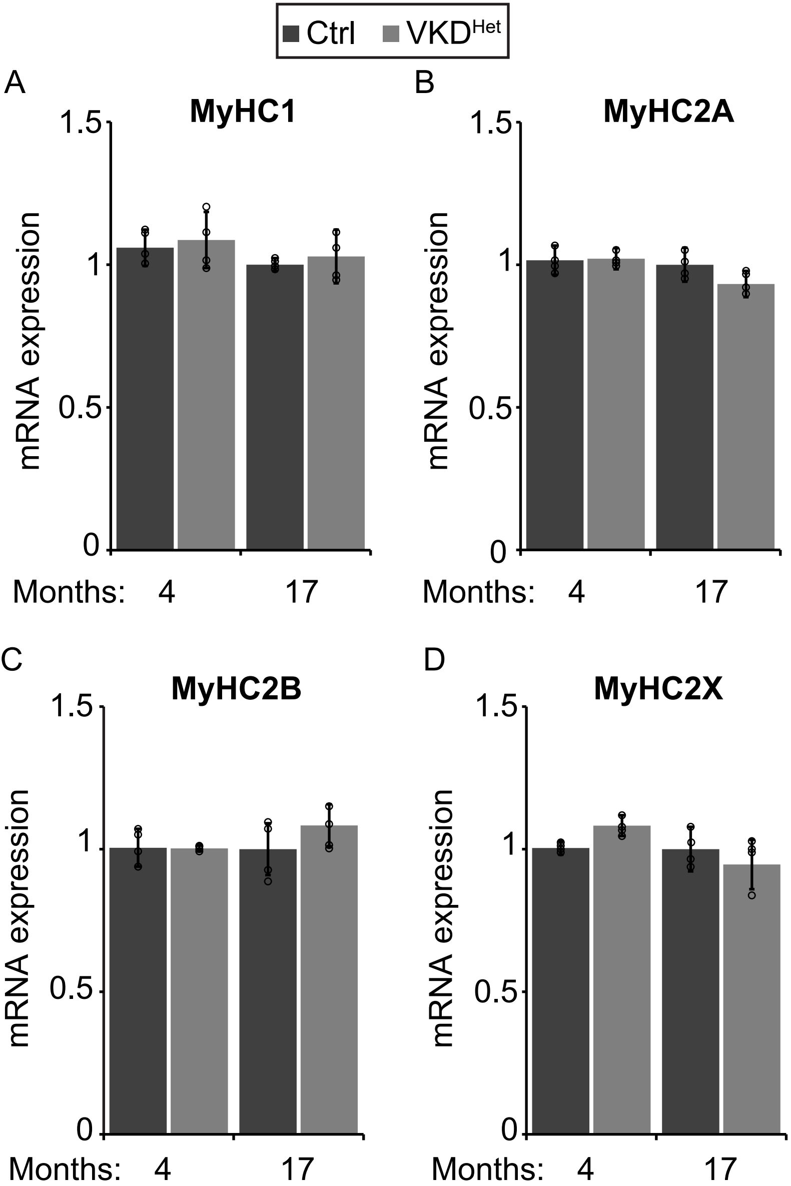

Ctrl
VKD^Het
A
MyHC1
B
MyHC2A
C
MyHC2B
D
MyHC2X
mRNA expression
1.5
1
0.5
0
Months: 4 17

Supplementary Figure 4. Normal molecular composition in aging VKD^{Het} muscle. There are no significant changes in the expression of MyHC1 (A), MyHC2A (B), MyHC2B (C), or MyHC2x (D) between control and VKD^{Het} mice at 4 or 17 months of age. Represented as mean ± SEM. Expression is normalized to GAPDH and relative to control. RNA isolated from TA muscles. Only male mice were used for these experiments. Control n=4; VKD^{Het} n=4 in each age group.
