# Supplementary material for: Attenuating Cholinergic Transmission Increases the Number of Satellite Cells and Preserves Muscle Mass in Old Age
Source: Front Aging Neurosci. 2019 Sep 24;11:262. doi: 10.3389/fnagi.2019.00262 (PMC6768977; doi:10.3389/fnagi.2019.00262)
Supplement: Supplementary file 2 [file Data_Sheet_2.PDF]

Table 1. PCR Primers

Gene	Fw (5'-3')	Rv (5'-3')
GAPDH	CCCACTCTTCCACCTTCGATG	GTCCACCACCCTGTTGCTGTAG
AChE	CTACACCACGGAGGAGAGGA	CTGGTTCTTCCAGTGCACCA
AChRγ	GCTCAGCTGCAAGTTGATCTC	CCTCCTGCTCCATCTCTGTC
AChRε	GCTGTGTGGATGCTGTGAAC	GCTGCCCAAAAACAGACATT
MuSK	CCACACTGCGTGGAATGAGC	GCTGCCCAAAAACAGACATT
LRP4	GGCAAAAAGCAGGAACTTGT	TCTACCCAGTGGCCAGAACT
Rapsyn	GTGCCATGGAGTGTTGTGAG	CGGTTTCCGATCTCAGTCAT
Dok7	GGGTACTGGGCTGGAGTCTT	TCGGACGATGCAGTCAAACA
CDK5	GCCAGACTATAAGCCCTACCC	GTCAGAGAAGTAGGGGTGCT
MyHC2A	GAGTGAGCAGAAGCGGAATGCT	GCGGAACTTGGATAGATTTGTG
MyHC2B	GCTAGTAACATGGAGGTCA	GCTCTTGCTCGGCCACTCT
MyHC2X	GCTAGTAACATGGAGGTCA	TAAGGCACTCTTGGCCTTTATC
Myogenin	GCACTGGAGTTCGGTCCCA	GTGATGCTGTCCACGATGGA
Foxo1	GAGTTAGTGAGCAGGCTACATTT	TTGGACTGCTCCTCAGTTCC
Atrogin-1	GCAGCAGCTGAATAGCATCCA	GGTGATCGTGAGGCCTTTGAA
Agrin-8	CTTTGATGGGCGGACCTACA	CGCTTTCTCAGCTGGGATCT
Agrin-11	CAGTGGGGGACCTAGAAACAC	TTTCAGGGCTCTCAGTCACAG
Agrin-19	CTTTGATGGGCGGACCTACA	AGTTTCAGGGGCTGGGATCT
ChAT	CCTGGATGGTCCAGGCACT	GTCATACCAACGATTCGCTCC
VAChT	GAGAGTACTTTGCCTGGGAGGA	GGCCACAGTAAGACCTCCCTTG
